# Simultaneous Determination of Three Coumarins in *Angelica dahurica* by ^1^H-qNMR Method: A Fast and Validated Method for Crude Drug Quality Control

**DOI:** 10.1155/2020/8987560

**Published:** 2020-03-24

**Authors:** Lan Yang, Qian Li, Yanmei Feng, Daiyu Qiu

**Affiliations:** Gansu Provincial Key Laboratory of Aridland Crop Science, College of Agronomy, Gansu Agricultural University, Lanzhou 730070, China

## Abstract

In this study, a quantitative ^1^H NMR method (^1^H-qNMR) for determining the contents of imperatorin, byakangelicin, and oxypeucedanin in *A. dahurica* in traditional Chinese medicine (TCM) has been established. Dried plant material was extracted exhaustively with methanol by an ultrasonication-assisted extraction method. The ^1^H-qNMR measurements were performed on a 600 -MHz spectrometer with hydroquinone as the internal standard reference in deuterated dimethyl sulfoxide (DMSO-d_6_) solvent. Quantification was carried out using the ^1^H resonance signals at 6.55 ppm for hydroquinone and 7.68, 7.38-7.39, and 6.38-6.39 ppm for imperatorin, byakangelicin, and oxypeucedanin, respectively. The linearity, limit of detection (LOD), limit of quantitation (LOQ), precision, reproducibility, stability, and recovery of the methodology were evaluated, and results were good. The newly developed method has been applied to determine the three coumarins in *A. dahurica*.

## 1. Introduction

*A. dahurica* is a perennial medicinal plant belonging to the dry root of the umbelliferous plant *A. dahurica* or *A. dahurica* var. formosana [1]. It was first published in Shennong's Herbal classics and listed as a middle product in China and has a long history of dual use of medicine and food [2]. *A. dahurica* is warm, fragrant, spicy, and slightly bitter, which has the effect of relieving stuffy nose, dissipating cold, expelling wind and acesodyne, removing dampness, clearing swelling and excluding pus, spasmolysis, analgesia, relieving asthma, anti-inflammatory immunomodulation, and skin whitening. In clinical practice, it is widely applied in the treatment of common cold, headache, nasal obstruction, rhinorrhea, toothache, leucorrhea, acne and carbuncles, rheumatism, and especially for the headache caused by wind-cold invading Yang and Yin with obvious curative effect [3–9]. Except for medicinal use, it is also widely used in food, health-care products, spices, skin care and beauty, daily chemical industry, and other aspects. In particular, its dry root can be used as an important condiment and spice to increase fragrance and taste, deodorize or remove odors, and increase appetite as well [10–12].

*A. dahurica* mainly contains coumarins, volatile oils, polysaccharides, and trace elements as the bioactive components [13]. Coumarins consist of imperatorin, isoimperatorin, bergapten, oxypeucedanin, byakangelicin, oxypeucedanin hydrate, and cnidilin [14–16]. These compounds possess multiple biological properties [17]. As imperatorin has been documented to have versatile pharmacological effects, for example anti-inflammatory, antineoplastic, hepatoprotective, photosensitive activity, and anticonvulsant [18, 19]. Isoimperatorin is a secondary plant metabolite that possesses multiple pharmacological properties, including fighting cancer-inducing substances, analgesic, and antiviral [20, 21]. Bergapten presents in the plants of Umbelliferae family and is widely used for its medicinal values such as anticoagulant, anti-inflammatory, and antiproliferative [22–24]. Byakangelicin is considered to be a natural potent inhibitor for aldose reductase and may be applied to the development of treatment for diabetic cataract [25]. Oxypeucedanin has been reported to have antimutagenic effects, cause uterus contraction, increase blood pressure, and have anticancer effects [26]. Therefore, the content of imperatorin, byakangelicin, oxypeucedanin, isoimperatorin, and bergapten in *A. dahurica* was determined in this study.

Until now, the HPLC method is often adopted for the determination of coumarins, which has disadvantages such as time consumption, complex sample pretreatment, and expensive standard substance needed to obtain. Therefore, it is practically significant to establish a rapid and reliable method for the determination of coumarins in *A. dahurica* [27–32]. As known to us, the quantitative ^1^H NMR method (^1^H-qNMR) can effectively characterize compound mixtures and quantify its constituents [33]. It has been widely and successfully applied for the quantitative analysis of chemical drugs, traditional Chinese medicine and plant extracts, body fluid samples, isomers, food, etc. [34, 35]. This method has the advantages of short measuring times, not requiring a high-purity reference standard for accurate quantification of the compounds of interest, the simplicity of the method, the ease of sample preparation, lower solvent usage, and being rapid [36–41].

In this study, a new method for the determination of imperatorin, byakangelicin, and oxypeucedanin in *A. dahurica* was established by using ^1^H-qNMR. Furthermore, the *A. dahurica* samples were analyzed, and it provides a theoretical and scientific basis for the quality control and evaluation of *A. dahurica*.

## 2. Experimental

### 2.1. General

The reference standard (RS) of imperatorin, oxypeucedanin, and isoimperatorin was purchased from Shanghai Yuanye Biotechnology Co., Ltd. (Shanghai, China). The reference standard (RS) of bergapten and byakangelicin was purchased from Chengdu Pufei De Biotechnology Co. Ltd. and Chengdu Ruifensi Biotechnology Co., Ltd. (Chengdu, China). The purities of all standard substances were greater than 98%. The internal standard (IS) hydroquinone was purchased from Shanghai Macklin Biochemical Co., Ltd. (Shanghai, China), and its purity was greater than 99%. Methanol was purchased from Tianjin Fuyu Fine Chemical Co. Ltd. (Tianjin, China). DMSO-d_6_ was purchased from Cambridge Isotope Laboratories, Inc.

The name and grade of the balance the authors used for weighing materials are electronic balance and II.

### 2.2. Plant Material

The plant material in the current study was bought from different origins of *A. dahurica* (China) from 20 to 30th October 2018 and identified by Professor Yuan Chen from the department of cultivation and identification of Chinese herbal medicine, Gansu Agricultural University. Prior to the analyses, the plant material was powdered using a blender and was sieved (40 order).

### 2.3. Preparation of Solution

#### 2.3.1. Preparation of Inner Standard Solution

The internal standard was dissolved in DMSO-d_6_.

#### 2.3.2. Preparation of Standard Solution

A mixed stock solution containing reference standards (imperatorin, byakangelicin, oxypeucedanin, isoimperatorin, and bergapten) was dissolved in the inner standard solution.

### 2.4. Sample Preparation for ^1^H-qNMR Analysis

Dried plant material (25 mg) was extracted exhaustively with methanol (2 × 125 mL) by using an ultrasonic extractor (40 min, 40°C), and the combined extracts were evaporated in a water bath and dried in a desiccator and then resolved in the inner standard solution (60 mg × 0.5 mL). The extraction was performed in triplicate for every plant material, and the NMR analysis was run in triplicate for every extract.

### 2.5. ^1^H NMR Spectroscopy

^1^H NMR spectra were acquired with a 600-MHz NMR spectrometer with a 5-mm probe. All data were processed using MestReNova software, unless otherwise stated. The following parameters were used for acquisition of spectra of a spectral width, 11904 Hz; acquisition time, 2.8 s; relaxation delay, 50 s; pulse width, 10 s; 16 scans; and temperature, 293.6 K. In addition, the influence of different relaxation delays 1 s, 5 s, 10 s, 15 s, 20 s, 50 s, and 100 s on the integral area was verified (Table 1). From the results of the integral area, there were almost no difference and no influence on the quantitative value. In this work, 50 s was chosen as the relaxation delay.

### 2.6. Validation

The analytical method was validated by the determination of the selectivity, linearity, limit of detection, limit of quantitation, precision, repeatability, stability, and recovery.

The selectivity was assessed by visual comparison between ^1^H NMR spectra of *A. dahurica* sample with the internal standard and reference standard.

The precision tests were performed by six replicate measurements of the reference standards with relative standard deviation (RSD) values considered as a measure of precision.

The repeatability was determined by six sample solutions (Suining sample, nonsulfur) with RSD values considered as a measure of repeatability.

To test the linearity, solutions with different concentrations (between 0.4 and 4 mg/mL) of the reference standards were prepared with the inner standard solution. The linearity was confirmed using the integral area ratio (*y*) and the mass ratio (*x*) of the standard and internal standard.

The limit of detection and quantitation can be determined by LOD = 3.3*σ*/s, LOQ = 10*σ*/s. *σ* shows deviation of the *y*-intercept of the nonzero intercept linear regression curve, and *s* shows the slope of the nonzero intercept linear regression curve.

The stability was determined by the same sample (Suining sample, nonsulfur) within 24 h with (RSD) values considered as a measure of stability.

Six samples of the tested content were added into the control solution of imperatorin, byakangelicin, and oxypeucedanin. The content of three coumarins in *A. dahurica* was determined using the developed method.

## 3. Results and Discussion

### 3.1. Selection of Solvent and Internal Standard

**Figure 1:**
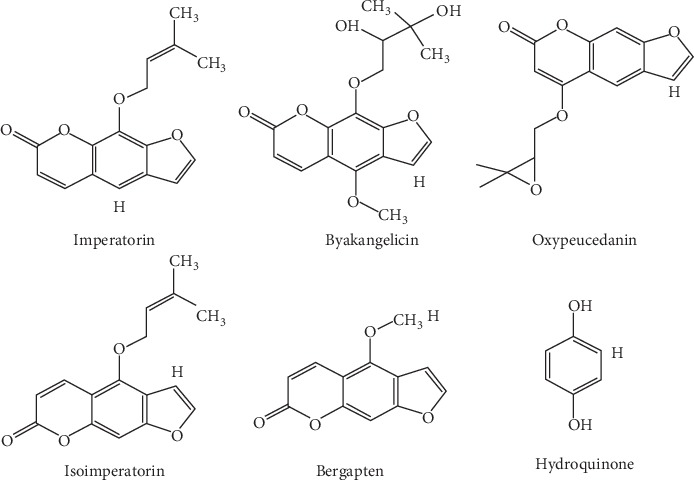
Structures of the internal standard and the reference standard.

**Figure 2:**
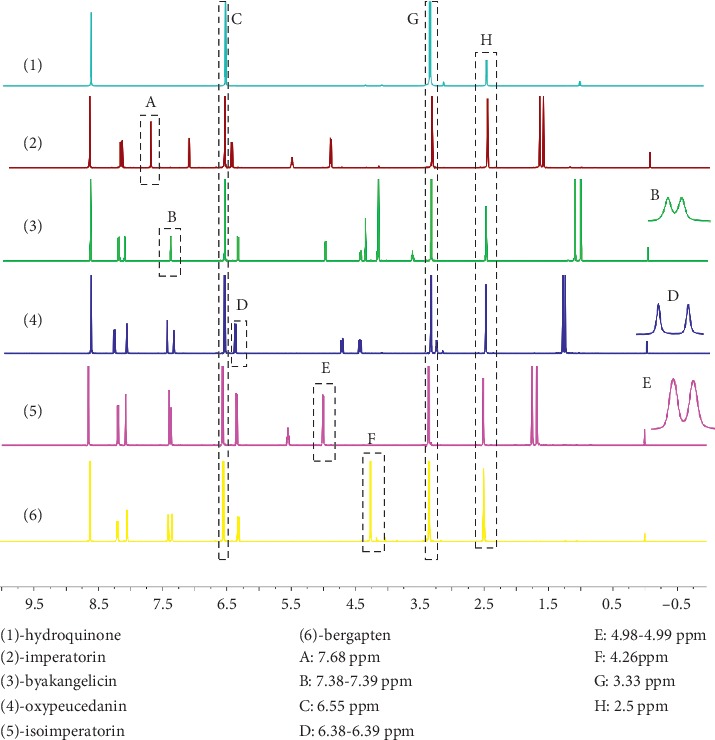
^1^H NMR spectrum of the internal standard and the reference standard in the DMSO-d_6_ solvent: (1) hydroquinone, (2) imperatorin, (3) byakangelicin, (4) oxypeucedanin, (5) isoimperatorin, and (6) bergapten; (A) 7.68 ppm, (B) 7.38-7.39 ppm, (C) 6.55 ppm, (D) 6.38-6.39 ppm, (E) 4.98-4.99 ppm, (F) 4.26 ppm, (G) 3.33 ppm, and (H) 2.5 ppm.

The suitable deuterium-substituted solvent should have good solubility for the samples and the internal standard. The internal standard should have a sharp single peak, which is easy to recognize, and the spectrum peak should not overlap with the peak to be tested. Through the preliminary experiment, DMSO-d_6_ was selected as the solvent and hydroquinone was selected as the internal standard. Quantification was carried out using the signals at 6.55, 7.68, 7.38-7.39, 6.38-6.39, 4.98-4.99, 4.26, 3.33, and 2.5 ppm for hydroquinone, imperatorin, byakangelicin, oxypeucedanin, isoimperatorin bergapten, H_2_O, and DMSO-d_6_ (standard reference), respectively (Figures 1 and 2).

### 3.2. Validation Studies

Validation of the developed procedure was performed in terms of selectivity, linearity, precision, repeatability, stability, and recovery.

Assignments were verified by comparison with the spectra of the reference standards. Signals of the quantified compounds selected for integration did not overlap with the signals from the same molecule-related constituents, solvents, or the internal standard.

The coefficient of determination (*R*^2^) obtained from the calibration curve construction (the integral area ratio and the mass ratio of the standard and internal standard) was 0.9994, 0.9992, and 0.9992 for imperatorin, byakangelicin, and oxypeucedanin, respectively. Therefore, the constructed analytical curves presented a satisfactory linearity (Table 2).

The calculation shows that the detection and quantitation limit were 0.173 mg/mL and 0.524 mg/mL for imperatorin, 0.124 mg/mL and 0.376 mg/mL for byakangelicin, and 0.149 mg/mL and 0.452 mg/mL for oxypeucedanin, respectively.

In this study, the precision and repeatability of method are good, and the sample solutions were stable within 24 h. The results are shown in Table 3.

The recovery of the three coumarins was good in this work, which is shown in Table 4.

### 3.3. Quantitative Results

**Figure 3:**
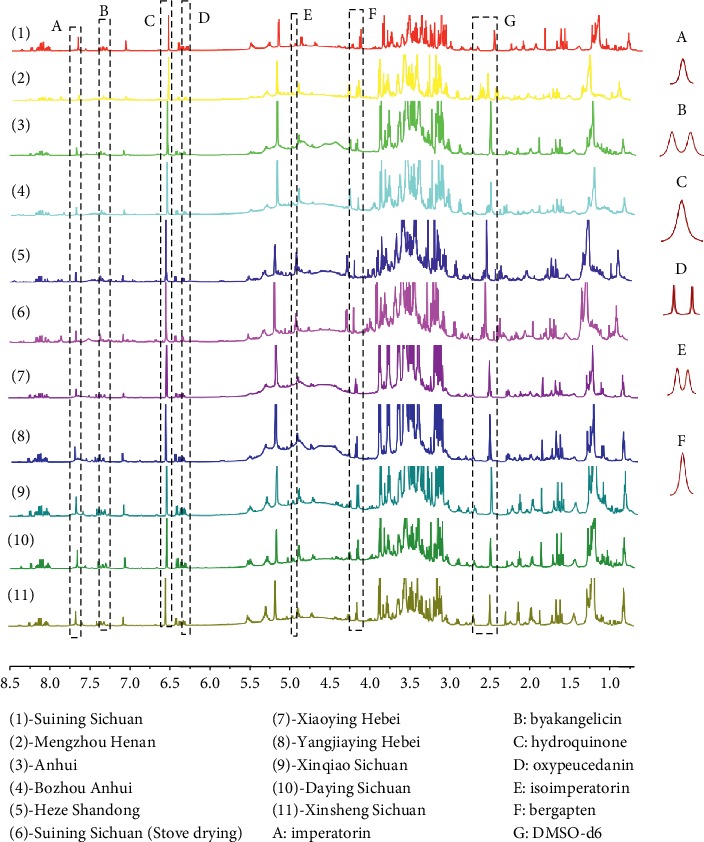
^1^H NMR spectrum of *A. dahurica* in the DMSO-d_6_ solvent: (1) Suining Sichuan, (2) Mengzhou Henan, (3) Anhui, (4) Bozhou Anhui, (5) Heze Shandong, (6) Suining Sichuan (stove drying), (7) Xiaoying Hebei, (8) Yangjiaying Hebei, (9) Xinqiao Sichuan, (10)- Daying Sichuan, and (11) Xinsheng Sichuan; (A) imperatorin, (B) byakangelicin, (C) hydroquinone, (D) oxypeucedanin, (E) isoimperatorin, (F) bergapten, and (G) DMSO-d_6_.

Using the developed method, the content of three coumarins in *A. dahurica* was determined by using ^1^H-qNMR for the first time (Table 5 and Figure 3).

As can be seen from Figure 3, the base lines around the signals of isoimperatorin (*E*) and bergapten (F) were not flat, so we did not use the signals at 4.98-4.99 and 4.26 ppm for qNMR measurement. But it can be determined that they are the signal peaks of isoimperatorin and bergapten.

## 4. Conclusion

In this study, the ^1^H-qNMR methodology was developed for determining the content of three coumarins in *A. dahurica*. This work provided a fast, simple, and validated method for the quality control of *A. dahurica*.

## Figures and Tables

**Table 1:** Effect of relaxation delay on the integral area.

Relaxation delay (s)	1	5	10	20	50	100
Integral area	0.71	0.7	0.68	0.71	0.69	0.69

**Table 2:** Standard curves of three components in *A. dahurica*.

Compound	Regression equation	*r*	Linearity (mg)
Imperatorin	*Y* = 0.1081*X* − 0.014	0.9995	0.2∼2
Byakangelicin	*Y* = 0.08*X* − 0.0065	0.9992	0.2∼2
Oxypeucedanin	*Y* = 0.1216*X* − 0.0147	0.9991	0.2∼2

**Table 3:** Precision, repeatability, and stability of three coumarins in *A. dahurica* by using the ^1^H-qNMR method (unit: %).

Component	Precision	Repeatability	Stability
Imperatorin	0.848	1.258	1.639
Byakangelicin	0.751	1.892	1.692
Oxypeucedanin	0.675	1.202	1.263

**Table 4:** Recovery (%) of the coumarins in *A. dahurica* by the using the ^1^H-qNMR method.

Component	Content for the sample (mg)	Standard addition (mg)	Experimental value (mg)	Recovery (%)	Average recover (%)	RSD (%)
Imperatorin	1.214	1.217	2.422	99.297	97.504	1.300
	1.214	1.217	2.406	97.952		
	1.214	1.217	2.406	97.952		
	1.214	1.217	2.373	95.263		
	1.214	1.217	2.373	95.263		
	1.214	1.217	2.406	97.952		

Byakangelicin	0.675	0.700	1.377	100.263	98.817	1.463
	0.675	0.700	1.356	97.371		
	0.675	0.700	1.356	97.371		
	0.675	0.700	1.377	100.263		
	0.675	0.700	1.356	97.371		
	0.675	0.700	1.356	97.371		

Oxypeucedanin	0.968	1.000	1.907	93.904	93.038	0.932
	0.968	1.000	1.889	92.171		
	0.968	1.000	1.907	93.904		
	0.968	1.000	1.889	92.171		
	0.968	1.000	1.889	92.171		
	0.968	1.000	1.924	95.638		

**Table 5:** Content (%) of three coumarins in *A. dahurica* by using the ^1^H-qNMR (unit: %).

Sample	Job operation	Component		
		Imperatorin	Byakangelicin	Oxypeucedanin
Suining Sichuan	—	0.239	0.133	0.191
Suining Sichuan	Stove drying	0.132	0.175	0.219
Xinqiao Sichuan	—	0.195	0.120	0.192
Daying Sichuan	—	0.221	0.117	0.173
Xinsheng Sichuan	—	0.207	0.125	0.186
Anhui	—	0.208	0.257	0.234
Bozhou Anhui	Stove drying	0.191	0.287	0.353
Xiaoying Hebei	Sun drying	0.363	0.236	0.337
Yangjiaying Hebei	Sun drying	0.392	0.315	0.335
Mengzhou Henan	Stove drying	0.093	0.219	0.190
Heze Shandong	Stove drying	0.132	0.187	0.217

“—” denotes unknown.

## Data Availability

The original 1HNMR spectral data and the analysis method of the data used to support the findings of this study are available from the corresponding authors upon request.
